# Editorial for the Special Issue “Characterization of Nanomaterials: Selected Papers from 6th Dresden Nanoanalysis Symposium”

**DOI:** 10.3390/nano9111527

**Published:** 2019-10-27

**Authors:** Ehrenfried Zschech, Robert Sinclair, Rodrigo Martins, Marco Sebastiani, Sabrina Sartori

**Affiliations:** 1 Fraunhofer Institute for Ceramic Technologies and Systems (IKTS), Microelectronic Materials and Nanoanalysis, 01109 Dresden, Germany; 2 Department of Materials Science and Engineering, Stanford University, Stanford, CA 94305, USA; bobsinc@stanford.edu; 3 i3N/CENIMAT, Department of Materials Science, Faculty of Science and Technology, Universidade NOVA de Lisboa and CEMOP/UNINOVA, Campus de Caparica, 2829-516 Caparica, Portugal; rfpm@fct.unl.pt; 4 Engineering Department, Università degli studi Roma Tre, via dellaVasca Navale, 79, 00146 Rome, Italy; seba@uniroma3.it; 5 Department of Technology Systems, University of Oslo, NO-2027 Kjeller, Norway; sabrina.sartori@its.uio.no

More than ever before, materials-driven product innovations in industry and shorter time-to-market introductions for new products require high advancement rates and a tight coupling between research, development and manufacturing. This approach, where scientists and engineers from industry and research institutes work together, includes sustained progress in materials science and engineering, and in materials and process characterization. Analytical techniques and respective tools, particularly to investigate nanomaterials, are considered to be fundamental drivers for innovation in industry. 

This Special Issue “Characterization of Nanomaterials” collects nine selected papers presented at the 6th Dresden Nanoanalysis Symposium, held at Fraunhofer Institute for Ceramic Technologies and Systems in Dresden, Germany, on 31 August 2018. The Dresden Nanoanalysis Symposium was organized by the Dresden Fraunhofer Cluster Nanoanalysis (DFCNA), supported by the European Materials Research Society (E-MRS) and the European Materials Characterization Council (EMCC). Following the specific motto of this annual symposium “Materials challenges—Micro- and nanoscale characterization”, it covered various topics of nanoscale materials characterization along the whole value and innovation chain, from fundamental research up to industrial applications. It brought about 100 scientists and engineers together from universities, research institutions, equipment manufacturers, and industrial end-users. New results in disruptive nanoanalysis techniques were reported in several talks and in the poster sessions, and novel solutions in the field of nanoscale materials characterization for process and quality control were shown. 

The scope of this Special Issue is to provide an overview of the current status, recent developments and research activities in the field of nanoscale materials characterization, with a particular emphasis on future scenarios. Primarily, analytical techniques for the characterization of thin films and nanostructures [1,2,3,4,5,6,7,8,9] are discussed, including modeling and simulation [9]. Particular techniques for materials characterization are 3D time-of-flight secondary ion mass spectroscopy [1], in situ X-ray diffraction [6] and electron backscatter diffraction [8]. Several papers cover advanced nanostructured materials for future electronic devices and sensors [2,3,5,7]. In addition, degradation processes in battery electrodes [4] and the growth kinetics of intermetallic phases [8] are addressed. We anticipate this Special Issue to be accessible to a wide audience, as it explores not only methodical aspects of nanoscale materials characterization, but also materials synthesis, fabrication of devices and applications.

The first paper describes the application of time-of-flight secondary ion mass spectroscopy (ToF-SIMS) in 3D microstructures, specifically so-called through-silicon vias, i.e., high-aspect ratio metal contacts needed for connecting 3D-stacked microchips [1]. This novel methodical approach provides several advantages compared to other frequently used techniques such as electron microscopy. The authors used data image slicing of the 3D ToF-SIMS analysis to study the uniformity of the silicon dopant concentration in atomic layer deposited (ALD) HfO_2_ thin films.

Barros et al. report on the role of structure and composition on the performances of p-type SnO_x_ thin-field transistors (TFTs) with a bottom gate configuration deposited by radio frequency magnetron sputtering at room temperature, followed by a post-annealing step [2]. X-ray diffraction (XRD) and Mössbauer spectroscopy allow the authors to identify the best phases/compositions and thicknesses (around 12 nm) to fabricate p-type TFTs. Moreover, the authors provide an overview that presents latest developments in SnO*_x_* TFT processing.

Studying multi-level resistive switching characteristics of a Cu_2_O/Al_2_O_3_ bilayer device, other authors found that an oxidation state gradient in copper oxide induced by the fabrication process plays a dominant role in defining multiple resistance states [3]. The highly conductive grain boundaries of copper oxide—an unusual property for an oxide semiconductor—are discussed for the first time regarding their role in the resistive switching mechanism.

The authors of paper [4] study materials aging in nickel manganese cobalt (NMC) oxides and related lifetime-reducing degradation processes in cathodes of lithium-ion batteries. Sieber et al. present an approach to recover NMC particles from lithium-ion battery cathodes while preserving their chemical and morphological properties, with a minimal use of chemicals. The key task was the separation of the cathode coating layer consisting of NMC, an organic binder, and carbon black, from the Al substrate foil. This can be performed in water under strong agitation to support the slow detachment process. The authors mitigate negative effects such as dissolving the Al substrate foil and Al(OH)_3_ precipitation using pH-adjusted solutions with sufficiently high buffer capacities.

Dobosz et al. study the oxide layer formed on the surface of the Ga–Sn–Zn eutectic alloy using atomic force microscopy (AFM), X-ray photoelectron spectroscopy (XPS) and transmission electron microscopy (TEM) [5]. The authors find that it is possible to obtain nanocrystalline oxide layers that contain about 90 at-% gallium with some additions of tin and zinc. 

Another paper describes the synthesis of upconverter nanostructures composed of an yttrium oxide host matrix co-doped with ytterbium and europium, i.e., Y_2_O_3_:Yb^3+^/Eu^3+^ [6]. These nanostructures were characterized by X-ray diffraction (XRD), scanning transmission electron microscopy (STEM) and scanning electron microscopy (SEM). The acetic-based nanostructures result in nanosheets with a thickness of about 50 nm, while hydrochloric and nitric-based ones result in sphere-shaped nanostructures. 

Carvalho et al. report about printed and flexible inorganic electrolyte-gated transistors (EGTs) on paper, with a channel layer based on interconnected zinc oxide (ZnO) nanoparticles [7]. The ZnO nanoparticles were dispersed by ethyl cellulose (EC), an eco-friendly binder. Fully printed devices on glass substrates using a composite solid polymer electrolyte as gate dielectrics exhibit a saturation mobility above 5 cm^2^ V^−1^ s^−1^ after annealing at 350 °C. The authors optimize the nanoparticle content in the ink, resulting in the formation of a ZnO channel layer at a maximum annealing temperature of 150 °C, which is compatible with paper substrates. 

The reactivity and kinetics of intermetallic phase growth in the Ni/Al/Ni system for nickel substrates while varying size and shape of the Ni grains are studied by Kwiecien et al. [8]. The sequence of the formation of particular intermetallic phases is determined using scanning electron microscopy (SEM), energy dispersive X-ray spectroscopy (EDX), and electron backscattered diffraction (EBSD) as well as transmission electron microscopy (TEM). 

Finally, this Special Issue features a study of the influence of nanoscale residual stress depth gradients on the nanomechanical behavior and adhesion energy of aluminum nitride (AlN) and Al/AlN thin films sputtered on a (100) silicon substrate [9]. Using a focused ion beam (FIB) incremental ring-core method, the residual stress depth gradient is assessed in the films. The adhesion energy was then quantified using a nanoindentation-based model. The authors show that an additional Al bond layer and inhomogeneous residual stresses increase the tensile stress at the coating/substrate interface and, consequently, negatively affect the adhesion of AlN to a substrate such as silicon.

The papers collected here reflect the existing widespread interest in materials characterization for materials research, development, and innovation, but also for process and quality control in industry, and provide an insight particularly into the directions in which new developments in characterization techniques are currently headed. In this Special Issue, material transitions that are necessary to improve the performance and to maintain the reliability of products for applications in microelectronics and energy storage are highlighted.

As lively discussed and consistently found during the symposium, research and development in materials characterization techniques are increasingly needed for modern materials science, for innovation in high-tech branches and to guarantee the functionality, performance and reliability of advanced products. We hope that this Special Issue will stimulate fruitful discussions and co-operation between experts in academia and industry, who are working in the field of advanced materials characterization. 
